# Randomized controlled trials: who fails run-in?

**DOI:** 10.1186/s13063-016-1451-9

**Published:** 2016-07-29

**Authors:** Judy R. Rees, Leila A. Mott, Elizabeth L. Barry, John A. Baron, Jane C. Figueiredo, Douglas J. Robertson, Robert S. Bresalier, Janet L. Peacock

**Affiliations:** 1 Department of Epidemiology, Geisel School of Medicine at Dartmouth, HB 7927, Dartmouth-Hitchcock Medical Center 1 Medical Center Drive, Lebanon, NH 03756 USA; 2 Department of Medicine, University of North Carolina School of Medicine, CB #7555, 4160-A Bioinformatics Bldg., 130 Mason Farm Rd, Chapel Hill, NC 27599 USA; 3 Department of Preventive Medicine, Keck School of Medicine, University of Southern California, Los Angeles, CA 90089 USA; 4 Department of Medicine, VA Medical Center, White River Junction, VT and Geisel School of Medicine at Dartmouth, Hanover, NH 03755 USA; 5 Department of Gastroenterology, Hepatology and Nutrition, The University of Texas MD Anderson Cancer Center, Houston, TX USA; 6 Division of Health and Social Care Research, King’s College London, London, UK and NIHR Biomedical Research Centre at Guy’s and St Thomas’ NHS Foundation Trust and King’s College, London, UK

**Keywords:** Randomized controlled trials, Run-in, Adherence, Generalizability

## Abstract

**Background:**

Early identification of participants at risk of run-in failure (RIF) may present opportunities to improve trial efficiency and generalizability.

**Methods:**

We conducted a partial factorial-design, randomized, controlled trial of calcium and vitamin D to prevent colorectal adenoma recurrence at 11 centers in the United States. At baseline, participants completed two self-administered questionnaires (SAQs) and a questionnaire administered by staff. Participants in the full factorial randomization (calcium, vitamin D, both, or neither) received a placebo during a 3-month single-blinded run-in; women electing to take calcium enrolled in a two-group randomization (calcium with vitamin D, or calcium alone) and received calcium during the run-in. Using logistic regression models, we examined baseline factors associated with RIF in three subgroups: men (*N* = 1606) and women (*N* = 301) in the full factorial randomization and women in the two-group randomization (*N* = 666).

**Results:**

Overall, 314/2573 (12 %) participants failed run-in; 211 (67 %) took fewer than 80 % of their tablets (poor adherence), and 103 (33 %) withdrew or were uncooperative. In multivariable models, 8- to 13-fold variation was seen by study center in odds of RIF risk in the two largest groups. In men, RIF decreased with age (adjusted odds ratio [OR] per 5 years 0.85 [95 % confidence interval, CI; 0.76–0.96]) and was associated with being single (OR 1.65 [95 % CI; 1.10–2.47]), not graduating from high school (OR 2.77 [95 % CI; 1.58–4.85]), and missing SAQ data (OR 1.97 [1.40–2.76]). Among women, RIF was associated primarily with health-related factors; RIF risk was lower with higher physical health score (OR 0.73 [95 % CI; 0.62–0.86]) and baseline multivitamin use (OR 0.44 [95 % CI; 0.26–0.75]). Women in the 5-year colonoscopy surveillance interval were at greater risk of RIF than those with 3-year follow-up (OR 1.91 [95 % CI; 1.08–3.37]), and the number of prescription medicines taken was also positively correlated with RIF (*p* = 0.03). Perceived toxicities during run-in were associated with 12- to 29-fold significantly increased odds of RIF.

**Conclusions:**

There were few common baseline predictors of run-in failure in the three randomization groups. However, heterogeneity in run-in failure associated with study center, and missing SAQ data reflect potential opportunities for intervention to improve trial efficiency and retention.

**Trial registration:**

ClinicalTrials.gov: NCT00153816. Registered September 2005.

**Electronic supplementary material:**

The online version of this article (doi:10.1186/s13063-016-1451-9) contains supplementary material, which is available to authorized users.

## Background

The run-in period of a trial is a participatory phase between enrollment and randomization to determine participants’ eligibility to continue in the trial. At the end of run-in, participants are randomized only if they meet prespecified criteria. A common use of the run-in period is to identify and exclude individuals who are likely to adhere poorly to the trial protocol. When used appropriately, this helps to minimize dropouts after randomization [1] and maximize trial efficiency and statistical power in the estimation of efficacy, although it may impair external validity if run-in failures (RIFs) are systematically different than those retained in the trial. Other uses of a run-in period are to identify and remove “placebo responders,” to identify the best effective or tolerated dose for each participant, to select participants with a good clinical response to (or tolerance of) the active treatment [2], or to establish baseline measurements for comparison after the intervention has been applied [3]. Such uses of a run-in period may introduce various forms of bias [4]. The run-in also offers time before randomization for participants to change their minds about taking part and for investigators to verify eligibility, for example, through review of enrollment blood test results and medical records.

In a trial whose placebo run-in is specifically designed to assess adherence and select adherent participants, participants may be largely responsible for their ineligibility, e.g., due to poor pill-taking or changing their mind about participation; in other cases they may have been good candidates to take part if factors beyond their control had not intervened, e.g., abnormal blood test results. Simplistically, we can think of these groups as voluntary and involuntary RIFs, respectively. An understanding of the characteristics associated with voluntary RIF may increase efficiency in trial planning, help determine enrollment targets for subgroups at risk of failing run-in, or be used to identify participants who might be retained in the trial using motivational strategies.

During a multicenter, randomized, placebo-controlled trial of calcium and vitamin D in the chemoprevention of colorectal adenoma recurrence [5], we examined the characteristics of participants who became “voluntary run-in failures” after an approximately 3-month single-blinded placebo run-in period.

## Methods

We conducted a randomized, double-blinded, placebo-controlled multicenter trial of daily supplementation with 1000 IU vitamin D_3_ and/or 1200 mg elemental calcium as calcium carbonate, for the prevention of large bowel adenomas [5]. Participants were recruited between 2004 and 2008 at 11 clinical centers in the continental United States and Puerto Rico following a complete colonoscopy, during which at least one colorectal adenoma was removed and none remained after the procedure. Eligible participants aged 45–75 years were in good general health and had no contraindications to calcium or vitamin D, no familial colorectal cancer syndromes, and no history of serious gastrointestinal disease. All participants provided written informed consent; the research was approved by the Committee for the Protection of Human Subjects at Dartmouth College and by Institutional Review Boards (IRBs) at each clinical center (see Additional file 1 for the list). The trial is reported according to the Consolidated Standards of Reporting Trials CONSORT (see Additional file 2 for the CONSORT checklist).

### Enrollment and run-in

During the informed consent process at enrollment, participants received an explanation about the study procedures, including randomized allocation to the study agents, and were provided a copy of the signed and dated informed consent form. Participants were mailed the SF-36 short form health survey and food frequency questionnaires to complete at home and bring to the enrollment interview. During a 2- to 3-hour in-person interview, they completed a detailed intake questionnaire including questions on their beliefs about the study interventions and the health effects of vitamin D: *preference*: “If you could choose, which kind of pill would you like to receive during the study?”; *efficacy beliefs*: “How likely do you think it is that vitamin D supplements [are helpful in preventing colon polyps]/[improve general health]/[improve pain in bones and joints]/[improve mood or depression]/[cause constipation]?”; and *allocation belief*: “If you were to place a bet, which pill would you bet you’ll be given during the trial?”. The latter question was poorly received by many participants and was removed from the survey after several months of use.

Participants were given a 7-day pill dispenser and their first bottle of study tablets containing placebo tablets, or calcium tablets for those in the two-group randomization, dispensed in a single-blinded manner. Information about the contents of the medicine bottle was shown on the label as: “This bottle contains one of the following: calcium, vitamin D, vitamin D + calcium, or placebo” or, for the two-group randomization, “This bottle contains one of the following: calcium, or vitamin D + calcium”, with instructions to take one tablet twice a day with food. The granulation and coating of the placebo tablets were composed of inactive compounds such as lactose (no more than 600 mg), cellulose, polymers, and minerals. All study tablets were manufactured for the study and looked similar. Participants were asked to take the first tablet during the enrollment interview; in some cases the first tablet was taken at home, and the participant was asked to confirm this by postcard or telephone. Participants were also given a study brochure, including general instructions on taking the tablets and specific instructions on staggered administration if they were taking medicines that might interact with calcium. Participants were asked to discontinue any personal calcium- or vitamin D-containing supplements and, for the duration of the trial, were offered a supply of multivitamins that excluded those ingredients. The label on the multivitamin bottle identified these as “Multivitamin supplement” and included a complete list of ingredients. Participants were counseled on the major dietary sources of calcium and vitamin D and were asked to avoid regularly consuming large amounts of such foods.

Blood was drawn at enrollment and tested for calcium, 25-hydroxyvitamin D (25-OH D), and creatinine levels. Where possible, an appointment was scheduled for a telephone interview in 2 to 3 months’ time. Participants received $100 at completion of the enrollment interview. Study coordinators and investigators were aware of the placebo (or calcium, in the two-group randomization) run-in, but participants were not informed about the run-in period. During run-in, medical records were reviewed and blood test results became available; individuals found to have disqualifying medical conditions or abnormal blood results (including serum 25-OH D levels <12 ng/ml) were ineligible for randomization. Throughout the trial, perceived toxicity reports were completed when participants reported symptoms that they attributed to the study tablets.

### Randomization

After a single-blinded run-in period of approximately 3 months (56–84 days), coordinators confirmed that the eligibility criteria necessary for randomization had been met, including a one-hour telephone interview to determine patient-reported eligibility criteria. They obtained the self-reported number of tablets left in the participant’s bottle, calculated the percentage of tablets taken, and disqualified from randomization anyone with self-reported adherence below 80 %. RIFs were generally discontinued from the study before completion of the full questionnaire. In rare cases, coordinators used their discretion, e.g., to retain a non-adherent participant who had misunderstood the dose during run-in, or to exclude a participant with unusual circumstances unlikely to adversely affect participation.

Eligible participants who confirmed their ongoing commitment to the study were block-randomized to treatment in a double-blinded manner, using a web-based random number generator, stratified by study center, sex, and colonoscopy interval (3 or 5 years). Those in the full factorial randomization were randomly assigned to one of four treatment groups (calcium, vitamin D, both, or placebo). Those in the two-group randomization (i.e., women who were not willing to forgo calcium supplementation) were randomized to vitamin D or placebo and were all provided with calcium. Study treatment was scheduled to continue until the surveillance colonoscopy either 3 or 5 years after the qualifying examination, according to recommendations by each participant’s gastroenterologist.

### Definitions: voluntary and involuntary run-in failures

RIFs were defined as individuals who enrolled and consented to participate in the trial but were not randomized after the run-in period. We defined “voluntary” RIFs as participants with some degree of control over the factors that prevented their randomization, i.e., those who declined to continue; those who took <80 % of their study tablets; and those who could not be reached for the telephone interview to be randomized. “Involuntary” RIFs were defined as participants whose removal from the trial was beyond their control, e.g., those whose safety blood tests were abnormal; those confirmed after enrollment as having a disqualifying medical condition; and others who changed residence after enrollment. Had the disqualifying medical information been available earlier, these participants would have been ineligible for enrollment. We focus on voluntary RIFs throughout this paper.

### Statistical methods

We reasoned that the determinants of voluntary RIF are likely to vary in different trials, depending on the type of intervention offered and the characteristics of participants who enroll in a particular trial. For this reason, a priori we chose to analyze three groups of participants separately to illustrate what might happen in parallel “trials” with minor differences in participant characteristics: (M4) men in the full factorial randomization; (F4) women in the full factorial randomization; and (F2) women in the two-group randomization, i.e., women who chose to take calcium. We reasoned that the predictors of RIF in a single, pooled analysis would lack external validity, whereas common factors identified independently in the three subgroups might generate more plausible hypotheses that could be tested in future, similar trials.

For each group of participants, we looked for univariate associations between baseline factors and RIF using chi-square, *t* test, and analysis of variance. All variables that had a *p* value <0.1 in univariate analyses were added to a ”full” logistic regression model. The final model was obtained by removing all variables in the full model with *p* > 0.05 in order to give a minimum set of variables that together contribute significantly to the model. If significant univariately, the reported perceived toxicity (the only post-enrollment factor examined) was added to the final model to allow us to assess how perceived toxicity influences the effect of the final model factors. For women in the two-group randomization, multivitamin use, calcium supplementation, and vitamin D supplementation were collinear, and we chose to include only multivitamin use in the full model.

#### Post hoc analyses

In general, the analysis plan and potential predictors of RIF were selected a priori; however, during the analysis we discovered substantial variability in RIF according to which study center the participant had been recruited. This was unexpected and led us to undertake several post hoc analyses. (1) First, we recognized other variables that deserved investigation and tested those in the models. These were: imperfect completion of the interviewer-administered enrollment questionnaire or the self-administered SF-36 and food frequency questionnaires; the participant’s willingness to set a date at enrollment for the next interview; the study experience of the enrolling staff member in prior polyp prevention studies (dichotomous) and the total number of participants they enrolled during the entire study. For logistical reasons, we were only able to measure this by summing enrollments retrospectively, understanding that total accrual did not reflect staff experience up to the point at which each individual was enrolled. The variables used in these post hoc analyses are identified as such in tables. (2) We reasoned that the observed effect of the study center may reflect a range of factors from regional differences among participants (e.g., education, race) to differences in methods used by study staff; we examined this possibility by building multivariable models without the center variable. (3) Finally, we investigated the importance of RIF rates at each center for long-term trial efficiency by assessing the proportion failing run-in at each center in relation to three measures of post-randomization adherence, using scatterplots and Kendall’s tau-b.

Odds ratios are presented with 95 % confidence intervals. A single *p* value for each categorical variable was estimated using a likelihood ratio test. Analyses were done using SAS (version 9) and STATA (version 14).

## Results

**Fig. 1:**
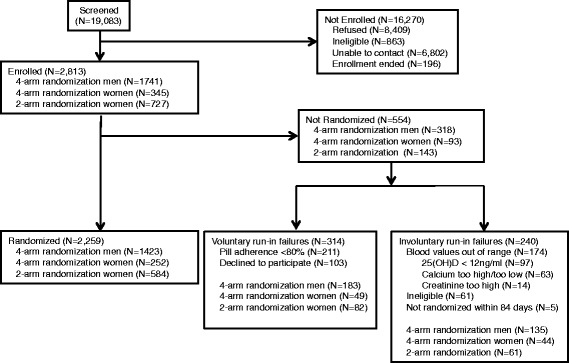
CONSORT flow diagram

Of 2813 enrollees, 240 (8.5 %) were excluded from randomization for reasons beyond their control such as out-of-range blood test results (involuntary RIFs, Fig. 1), leaving 2573 enrollees who were medically eligible for randomization. A further 314/2573 (12 %) were not randomized because of poor adherence or refusal to continue in the study (voluntary RIFs, Fig. 1). The RIF proportions among M4, F4, and F2 were 183/1606 (11 %), 49/301 (16 %), and 82/666 (12 %), respectively (Table 1). Of the 103 who declined to participate, 66 (21 % of RIFs, 2 % of those eligible) clearly stated that they did not want to continue in our study (15 because of a perceived toxicity [PT]), and 37 (12 % of RIFs) were uncooperative or could not be contacted. Two-thirds (*N* = 211) of RIFs (8 % of those eligible) were attributed to poor pill-taking adherence.

**Table 1:** Participant characteristics at enrollment for randomized participants and voluntary run-in failures

	Full factorial randomization						Two-group randomization		
	Men (*N* = 1606)			Women (*N* = 301)			Women (*N* = 666)		
	Randomized	Voluntary run-in failure	*p*	Randomized	Voluntary run-in failure	*p*	Randomized	Voluntary run-in failure	*p*
	*N* (%)	*N* (%)		*N* (%)	*N* (%)		*N* (%)	*N* (%)	
Total participants	1423 (89)	183 (11)		252 (84)	49 (16)		584 (88)	82 (12)	
PERSONAL FACTORS									
Age mean (SD)	58.8 (6.9)	57.5 (7.1)	0.01	56.6 (6.9)	57.5 (6.0)	0.41	56.8 (6.3)	57.3 (7.2)	0.59
Race			0.02^a^			0.04^a^			0.02^a^
White	1214 (90)	140 (10)		211 (85)	37 (15)		475 (90)	55 (10)	
Black	100 (83)	21 (17)		31 (86)	5 (14)		53 (78)	15 (22)	
Asian	35 (88)	5 (12)		1 (100)	0 (0)		15 (94)	1 (6.3)	
Native American	9 (100)	0 (0)		2 (67)	1 (33)		2 (100)	0 (0)	
Pacific Islander	1 (100)	0 (0)		1 (100)	0 (0)		0 (0)	0 (0)	
Multiple races	9 (75)	3 (25)		0 (0)	1 (100)		1 (100)	0 (0)	
Unknown/refused	55 (80)	14 (20)		6 (55)	5 (45)		38 (78)	11 (22)	
Hispanic ethnicity			<0.0001			0.06^a^			0.13
No	1337 (90)	154 (10)		243 (85)	44 (15)		530 (88)	70 (12)	
Yes	83 (77)	25 (23)		9 (64)	5 (36)		54 (82)	12 (18)	
Unknown/refused	3 (43)	4 (57)		0	0		0	0	
Marital status			0.001			0.04			0.04
Married/cohabitating	1232 (90)	140 (10)		182 (87)	28 (13)		421 (89)	50 (11)	
Single	191 (82)	41 (18)		70 (77)	21 (23)		162 (84)	32 (16)	
Unknown/refused	0	2 (100)		0	0		1 (100)	0	
Education			<0.0001			0.07			0.03
Did not graduate high school	63 (71)	26 (29)		16 (70)	7 (30)		46 (78)	13 (22)	
Graduated high school	196 (90)	21 (9.7)		41 (79)	11 (21)		83 (85)	15 (15)	
Any college education	1164 (90)	135 (10)		195 (86)	31 (14)		455 (89)	54 (11)	
HEALTH-RELATED FACTORS									
Smoking status			0.40			0.06			0.46
Never	664 (89)	85 (11)		156 (88)	22 (12)		374 (89)	47 (11)	
Former	629 (89)	75 (11)		71 (80)	18 (20)		150 (85)	26 (15)	
Current	130 (86)	22 (14)		25 (74)	9 (26)		60 (87)	9 (13)	
Unknown/refused	0	1 (100)		0	0		0	0	
Alcohol use			0.96			0.32			0.10
None	341 (90)	39 (10)		116 (81)	27 (19)		233 (85)	40 (15)	
≤1 drink/day	519 (90)	56 (9.7)		91 (88)	13 (12)		244 (91)	24 (9.0)	
>1 drink/day	453 (90)	51 (10)		26 (79)	7 (21)		71 (86)	12 (14)	
Unknown/refused	110 (75)	37 (25)		19 (90)	2 (9.5)		36 (86)	6 (14)	
Activity level			0.96			0.72			0.66
Low	318 (89)	41 (11)		63 (81)	15 (19)		152 (87)	23 (13)	
Moderate	424 (89)	52 (11)		86 (84)	17 (16)		202 (87)	29 (13)	
High	665 (89)	86 (11)		98 (85)	17 (15)		222 (90)	26 (10)	
missing	16 (80)	4 (20)		5 (100)	0		8 (67)	4 (33)	
Taking a multivitamin			0.04			0.59			0.001
No	650 (87)	97 (13)		149 (83)	31 (17)		191 (82)	42 (18)	
Yes	771 (90)	83 (9.7)		103 (85)	18 (15)		393 (91)	38 (8.8)	
Unknown/refused	2 (40)	3 (60)		0	0		0	2 (100)	
Taking calcium supplement			0.06			0.10			0.001
No	1321 (88)	174 (12)		221 (82)	47 (18)		273 (84)	53 (16)	
Yes	100 (94)	6 (5.7)		30 (94)	2 (6.3)		310 (92)	27 (8.0)	
Unknown/refused	2 (40)	3 (60)		1 (100)	0		1 (33)	2 (67)	
Taking vitamin D supplement			0.04			0.39^a^			0.0001
No	1360 (88)	178 (12)		229 (83)	47 (17)		370 (84)	68 (16)	
Yes	61 (97)	2 (3.2)		21 (91)	2 (8.7)		213 (95)	12 (5.3)	
Unknown/refused	2 (40)	3 (60)		2 (100)	0		1 (33)	2 (67)	
Number of prescription medications			0.27			0.02			0.77
0	307 (86)	50 (14)		29 (78)	8 (22)		84 (87)	13 (13)	
1	360 (90)	38 (9.6)		59 (89)	7 (11)		145 (90)	16 (10)	
2	273 (88)	36 (12)		51 (94)	3 (5.6)		114 (87)	17 (13)	
3+	483 (89)	59 (11)		113 (78)	31 (22)		241 (87)	36 (13)	
Experienced chronic fatigue in last year			0.66			0.41^a^			0.11
None	1357 (89)	171 (11)		219 (83)	44 (17)		531 (89)	69 (11)	
Some	51 (85)	9 (15)		23 (92)	2 (8.0)		40 (78)	11 (22)	
Severe	15 (88)	2 (12)		10 (77)	3 (23)		13 (87)	2 (13)	
Don’t know	0	1 (100)		0	0		0	0	
Experienced muscular pain in last year			0.53			0.05			0.33
None	1228 (89)	154 (11)		210 (83)	43 (17)		488 (89)	63 (11)	
Some	171 (88)	23 (12)		38 (93)	3 (7.3)		76 (85)	13 (15)	
Severe	23 (82)	5 (18)		4 (57)	3 (43)		20 (80)	5 (20)	
Don’t know	1 (50)	1 (50)		0	0		0	1 (100)	
Experienced muscular weakness in last year			0.09			0.03^a^			0.31
None	1358 (89)	167 (11)		234 (83)	47 (17)		534 (88)	72 (12)	
Some	54 (82)	12 (18)		16 (100)	0		38 (81)	9 (19)	
Severe	11 (79)	3 (21)		2 (50)	2 (50)		11 (92)	1 (8.3)	
Don’t know	0	1 (100)		0	0		1 (100)	0	
Experienced bone aches or pains in last year			0.11			0.42			0.56
None	1215 (89)	151 (11)		208 (85)	37 (15)		455 (88)	63 (12)	
Some	170 (89)	21 (11)		30 (77)	9 (23)		96 (89)	12 (11)	
Severe	38 (79)	10 (21)		14 (88)	2 (12)		33 (83)	7 (17)	
Don’t know	0	1 (100)		0	1 (100)		0	0	
SF-36 mental component summary measure, mean (SD)^b^	55.9 (6.0)	55.0 (7.3)	0.09	53.9 (7.5)	53.0 (10.4)	0.58	54.2 (6.9)	51.8 (8.0)	0.01
SF-36 physical component summary measure, mean (SD)^b^	53.9 (6.1)	53.1 (7.5)	0.65	53.5 (6.8)	51.7 (7.5)	0.12	52.8 (6.8)	49.3 (8.9)	0.0004
ADENOMA-RELATED FACTORS									
Number of baseline adenomas			0.29			0.53			0.48
0	5 (83)	1 (17)		0	0		0	1 (100)	
1	916 (88)	128 (12)		190 (83)	39 (17)		453 (88)	60 (12)	
2+	502 (90)	54 (9.7)		62 (86)	10 (14)		131 (86)	21 (14)	
Baseline advanced adenomas			0.73			0.98			0.12
No	1139 (89)	147 (11)		201 (84)	39 (16)		476 (87)	73 (13)	
Yes	253 (88)	35 (12)		51 (84)	10 (16)		103 (92)	9 (8.0)	
Unknown	31 (97)	1 (3.1)		0	0		5 (100)	0	
Family history of colorectal cancer			0.14			0.40			0.73
No	1080 (88)	142 (12)		200 (84)	39 (16)		454 (88)	60 (12)	
Yes	238 (92)	22 (8.5)		39 (89)	5 (11)		95 (87)	14 (13)	
Unknown/refused	105 (85)	19 (15)		13 (72)	5 (28)		35 (81)	8 (19)	
Colonoscopy surveillance interval			0.73			0.12			0.003
3 year	727 (88)	96 (12)		123 (87)	18 (13)		287 (92)	26 (8.3)	
5 year	696 (89)	87 (11)		129 (81)	31 (19)		297 (84)	56 (16)	
STUDY-RELATED FACTORS									
Study center			<0.0001			0.33^a^			<0.0001
A	227 (98)	5 (2.2)		29 (85)	5 (15)		86 (97)	3 (3.4)	
B	119 (97)	4 (3.3)		3 (100)	0		57 (95)	3 (5.0)	
C	75 (96)	3 (3.9)		16 (100)	0		45 (96)	2 (4.3)	
D	75 (90)	8 (9.6)		12 (92)	1 (7.7)		43 (96)	2 (4.4)	
E	242 (88)	33 (12)		59 (86)	10 (14)		64 (93)	5 (7.3)	
F	137 (87)	20 (13)		27 (87)	4 (13)		43 (66)	22 (34)	
G	143 (86)	23 (14)		32 (86)	5 (14)		62 (94)	4 (6.1)	
H	170 (85)	29 (15)		28 (80)	7 (20)		50 (82)	11 (18)	
I	123 (83)	25 (17)		31 (74)	11 (26)		73 (84)	14 (16)	
J	28 (80)	7 (20)		0	0		9 (82)	2 (18)	
K	84 (76)	26 (24)		15 (71)	6 (29)		52 (79)	14 (21)	
Taking prescription drug that requires staggered administration with study tablets			0.10			0.13			0.21
No	1310 (89)	162 (11)		204 (85)	35 (15)		472 (87)	71 (13)	
Yes	113 (84)	21 (16)		48 (77)	14 (23)		112 (91)	11 (8.9)	
Counseled at baseline to change diet			0.57			0.13^a^			0.09^a^
No	1381 (89)	178 (11)		250 (84)	47 (16)		578 (88)	78 (12)	
Yes	42 (91)	4 (8.7)		2 (50)	2 (50)		6 (67)	3 (33)	
Unknown	0	1 (100)		0	0		0	1 (100)	
Refused any questions during enrollment in-person questionnaire^c,d^			0.01^a^			1.00^a^			0.01^a^
No	1419 (89)	179 (11)		251 (84)	49 (16)		583 (88)	79 (12)	
Yes	4 (50)	4 (50)		1 (100)	0		1 (25)	3 (75)	
Answered ”Don’t know” to any questions during enrollment in-person questionnaire^c,d^			0.01			0.59^a^			0.05
No	1314 (89)	159 (11)		231 (84)	44 (16)		521 (89)	67 (11)	
Yes	109 (82)	24 (18)		21 (81)	5 (19)		63 (81)	15 (19)	
Refused any questions during enrollment self-administered questionnaires^c^			<0.0001			0.02			0.001
No	1084 (91)	103 (8.7)		225 (86)	38 (14)		517 (89)	62 (11)	
Yes	339 (81)	80 (19)		27 (71)	11 (29)		67 (77)	20 (23)	
Scheduled next interview during enrollment^c^			0.001			0.25			0.29
No	261 (83)	52 (17)		44 (79)	12 (21)		113 (85)	20 (15)	
Yes	1162 (90)	131 (10)		208 (85)	37 (15)		471 (88)	62 (12)	
Number of enrollments conducted by coordinator^c^			0.42			0.31			0.76
1–10	16 (80)	4 (20)		6 (100)	0		17 (94)	1 (5.6)	
11–50	170 (86)	27 (14)		28 (82)	6 (18)		86 (87)	13 (13)	
51–100	364 (89)	46 (11)		77 (89)	10 (11)		132 (86)	21 (14)	
>100	873 (89)	106 (11)		141 (81)	33 (19)		349 (88)	47 (12)	
Coordinator worked in a prior polyp prevention trial^c^			0.01			0.48			0.49
No	1034 (87)	144 (13)		211 (84)	39 (16)		413 (87)	61 (13)	
Yes	389 (92)	34 (8.0)		41 (80)	10 (20)		171 (89)	21 (11)	

Missing data are shown where values are not zero. *p* values are based on non-missing data, except for race

^a^Fisher exact test

^b^ *p* value from Kruskal-Wallis test because SF-36 scores are skewed

^c^Not a priori potential predictors

^d^Excludes questions asked as part of a skip pattern (*N* = 43 questions for men and 47 questions for women) and questions about beliefs in which “Don’t know” was an allowable response

Treating the three randomization groups as though they represent three separate study populations, the univariate analyses identified several variables that were associated with a higher proportion of RIF in each of the three groups: non-white race (overall proportions in RIF and randomized participants respectively, 17 % and 11 %), lower educational attainment (overall 27 % versus 11 %), being unmarried (overall 18 % versus 11 %), and failing to complete one or more questions in either of the self-administered questionnaires (overall 20 % versus 10 %) (Table 1). In all three groups, participants at the enrollment interview who confirmed a date and time for the next appointment were less likely to fail run-in (overall 11 % versus 17 %), but this was only statistically significant in the largest group, M4 (*p* < 0.001). Five additional factors were significantly associated with RIF in univariate analyses in two of the three groups: Hispanic ethnicity, study center, a lower SF-36 mental health score, non-use of multivitamins before the study, and answering “Don’t know” or refusing to answer one or more questions during the in-person enrollment interview.

Generally, participants’ preferences and beliefs about the properties of the study tablets did not substantially influence their probability of being randomized (Table 2). At study entry, 63 % of participants believed that calcium and vitamin D were very or somewhat likely to prevent colorectal polyps, 34 % did not know, and 3 % thought this unlikely. Overall, a majority of participants believed that calcium and vitamin D were likely to improve general health (86 %), improve pain in bones and joints (63 %), or improve mood (32 %); 20 % believed the study agents were likely to cause constipation. In univariate analyses, beliefs about the effectiveness of calcium and vitamin D and the baseline guess about allocation were not significantly associated with RIF. Although RIF risk tended to be lower in participants who would prefer to receive both calcium and vitamin D (overall 11 % versus 14 %), this was not statistically significant.

**Table 2:** Participant beliefs and voluntary run-in failure

	Full factorial randomization						Two-group randomization		
	Men (*N* = 1606)			Women (*N* = 301)			Women (*N* = 666)		
	Randomized	Voluntary run-in failure	*p*	Randomized	Voluntary run-in failure	*p*	Randomized	Voluntary run-in failure	*p*
	*N* (%)	*N* (%)		*N* (%)	*N* (%)		*N* (%)	*N* (%)	
	1423 (89)	183 (11)		252 (84)	49 (16)		584 (88)	82 (12)	
BASELINE FACTORS									
Preference: If you could choose, which kind of pill would you like to receive during the study?									
Calcium + vitamin D	854 (90)	96 (10)	0.08	174 (87)	27 (13)	0.10^a^	450 (88)	62 (12)	0.37
Calcium only	67 (83)	14 (17)		13 (65)	7 (35)		34 (81)	8 (19)	
Vitamin D only	63 (83)	13 (17)		8 (80)	2 (20)		N/A	N/A	
Placebo	32 (94)	2 (5.9)		4 (100)	0		N/A	N/A	
Don’t know/refused	407 (88)	57 (12)		53 (80)	13 (20)		99 (89)	12 (11)	
Missing	0	1 (100)		0	0		1 (100)	0	
Efficacy belief: How likely do you think it is that vitamin D supplements …									
Are helpful in preventing polyps			0.74			0.72			0.44
Very/somewhat likely	852 (89)	108 (11)		173 (83)	36 (17)		381 (87)	58 (13)	
Don’t know	530 (89)	67 (11)		70 (86)	11 (14)		182 (89)	23 (11)	
Very/somewhat unlikely	40 (85)	7 (15)		8 (80)	2 (20)		20 (95)	1 (4.8)	
Refused/missing	1 (50)	1 (50)		1 (100)	0		1 (100)	0	
Improve health			0.20			0.42^a^			0.30
Very/somewhat likely	1189 (89)	143 (11)		239 (84)	45 (16)		514 (87)	76 (13)	
Don’t know	177 (85)	31 (15)		10 (77)	3 (23)		56 (90)	6 (9.7)	
Very/somewhat unlikely	56 (88)	8 (13)		3 (75)	1 (25)		13 (100)	0	
Refused/missing	1 (50)	1 (50)		0	0		1 (100)	0	
Improve pain in bones and joints			0.59			0.63			0.18
Very/somewhat likely	902 (89)	109 (11)		176 (83)	37 (17)		347 (86)	57 (14)	
Don’t know	374 (88)	51 (12)		44 (85)	8 (15)		184 (91)	18 (8.9)	
Very/somewhat unlikely	145 (87)	22 (13)		32 (89)	4 (11)		52 (88)	7 (12)	
Refused/missing	2 (67)	1 (33)		0	0		1 (100)	0	
Improve mood			0.31			0.47			0.06
Very/somewhat likely	375 (87)	56 (13)		86 (83)	18 (17)		240 (84)	45 (16)	
Don’t know	632 (90)	71 (10)		89 (87)	13 (13)		245 (91)	25 (9.3)	
Very/somewhat unlikely	414 (88)	55 (12)		77 (81)	18 (19)		98 (89)	12 (11)	
Refused/missing	2 (67)	1 (33)		0	0		1 (100)	0	
Cause constipation			0.36			0.46			0.09
Very/somewhat likely	240 (86)	38 (14)		82 (87)	12 (13)		117 (82)	25 (18)	
Don’t know	669 (89)	85 (11)		91 (83)	18 (17)		282 (90)	33 (10)	
Very/somewhat unlikely	511 (90)	59 (10)		79 (81)	19 (19)		184 (88)	24 (12)	
Refused/missing	3 (75)	1 (25)		0	0		1 (100)	0	
Efficacy score^b^ mean (SD) compared with [randomized participants]	2.8 (2.6)	2.5 (2.9)	0.20	3.1 (2.5)	3.4 (2.7)	0.39	3.3 (2.6)	3.6 (2.9)	0.26
Efficacy score^b^			0.16			0.94^a^			0.81
−9 to −1 (benefit unlikely)	97 (85)	17 (15)		21 (88)	3 (13)		29 (88)	4 (12)	
0 (no benefit)	149 (85)	26 (15)		16 (80)	4 (20)		49 (89)	6 (11)	
1–5 (little benefit)	957 (90)	109 (10)		168 (84)	33 (16)		390 (88)	52 (12)	
6–10 (some benefit)	220 (88)	31 (12)		47 (84)	9 (16)		116 (85)	20 (15)	
Allocation belief: If you were to place a bet, which pill would you bet you’ll be given during the trial?									
Calcium + vitamin D	236 (90)	25 (9.6)	0.96	38 (83)	8 (17)	0.89^a^	116 (85)	21 (15)	0.73
Calcium only	102 (89)	13 (11)		18 (82)	4 (18)		95 (88)	13 (12)	
Vitamin D only	91 (90)	10 (9.9)		11 (79)	3 (21)		N/A	N/A	
Placebo	206 (89)	25 (11)		31 (79)	8 (21)		N/A	N/A	
Don’t know/refused	150 (91)	15 (9.1)		18 (90)	2 (10)		29 (88)	4 (12)	
Missing/not asked^c^	638 (87)	95 (13)		136 (85)	24 (15)		344 (89)	44 (11)	
POST-ENROLLMENT FACTOR									
Perceived toxicity during the run-in period^d^									
No	1412 (89)	169 (11)	<0.0001^a^	249 (85)	43 (15)	0.001^a^	581 (89)	73 (11)	<0.0001^a^
Yes	11 (44)	14 (56)		3 (33)	6 (67)		3 (25)	9 (75)	

Missing data are shown where values are not zero. *p* values are based on non-missing data

^a^Fisher exact test

^b^Efficacy score was calculated as follows: For the first four health outcomes, extremely likely scores 2; somewhat likely scores 1; don’t know (or refused or missing) scores 0; somewhat unlikely scores –1; extremely unlikely scores –2. For constipation, the scoring system is reversed, e.g., very likely to cause constipation scores –2. All five scores are summed to give an overall efficacy score

^c^The number of respondents is smaller because this question was removed from the questionnaire

^d^Perceived toxicities are defined as reports made by study coordinators on symptoms that participants attribute to the study tablets. These are identified either during the randomization questionnaire or if participants specifically contact study coordinators to report them

The multivariable models developed for the three groups had few similarities (Tables 3, 4, 5). Study center was significantly associated with RIF in M4 and F2, with >8-fold variation in odds of RIF among the 11 centers in M4, and >13-fold in F2. In M4, men who missed or refused any question in the self-administered SF-36 or food frequency questionnaires had more than twice the odds of failing run-in (adjusted odds ratio [OR] 1.97; 95 % confidence interval [CI] 1.40–2.76). Younger men were more likely to fail run-in (adjusted OR per 5 years of age 0.85; 95 % CI 0.76–0.96), as well as single or divorced men (adjusted OR 1.65; 95 % CI 1.10–2.47) or men who had not graduated high school (OR 2.77; 95 % CI 1.58–4.85). Among women in the full factorial randomization (F4), RIF was more likely in those reporting use of no prescription medicines or three or more (*p* = 0.03). Among women in the two-group randomization (F2), in addition to study center, RIF was inversely associated with regular use of multivitamins at baseline (adjusted OR 0.44; 95 % CI 0.26–0.75) and SF-36 Physical Component Summary (PCS) measure (adjusted OR 0.73; 95 % CI 0.62–0.86). Women in the 5-year colonoscopy cycle had almost twice the odds of run-in failure as those with 3-year recommended follow-up (adjusted OR 1.91; 95 % CI 1.08–3.37).

**Table 3:** Logistic regression models of voluntary run-in failure: full factorial study males

	Unadjusted OR (95 % CI)^a^	*p*	Final model: adjusted OR (95 % CI) C index = 0.73	*p*	Final model + perceived toxicity adjusted OR (95 % CI)^b^ C index = 0.75	*p*
Total participants			*N* = 1604		*N* = 1604	
Age (per 5 years)	0.87 (0.78–0.97)	0.01	0.85 (0.76–0.96)	0.01	0.84 (0.75–0.95)	0.01
Race		0.01				
White	Reference					
Black	1.82 (1.10–3.01)					
Other	1.29 (0.60–2.76)					
Unknown/refused	2.21 (1.20–4.07)					
Hispanic ethnicity		<0.0001				
No	Reference					
Yes	2.62 (1.62–4.22)					
Marital status		0.001		0.01		0.01
Single	1.89 (1.29–2.76)		1.65 (1.10–2.47)		1.73 (1.16–2.60)	
Married/cohabitating	Reference		Reference		Reference	
Education		<0.0001		0.001		0.004
Did not graduate high school	3.56 (2.18–5.81)		2.77 (1.58–4.85)		2.57 (1.45–4.57)	
Graduated high school	0.92 (0.57–1.50)		0.93 (0.56–1.55)		0.90 (0.53–1.51)	
Any college education	Reference		Reference		Reference	
Taking a multivitamin		0.04				
No	Reference					
Yes	0.72 (0.53–0.99)					
Taking calcium supplements		0.07				
No	Reference					
Yes	0.46 (0.20–1.05)					
Taking vitamin D supplements		0.06				
No	Reference					
Yes	0.25 (0.06–1.03)					
Experienced muscular weakness in last year		0.09				
None	Reference					
Some	1.81 (0.95–3.45)					
Severe	2.22 (0.61–8.03)					
SF-36 mental component summary measure (per 5 units)	0.90 (0.80–1.01)	0.08				
Study center		<0.0001		0.0003		0.001
A	Reference		Reference		Reference	
B	1.53 (0.40–5.79)		1.54 (0.40–5.89)		1.52 (0.40–5.84)	
C	1.82 (0.42–7.78)		1.99 (0.46–8.56)		1.62 (0.36–7.27)	
D	4.84 (1.54–15.25)		4.34 (1.36–13.86)		4.38 (1.37–14.02)	
E	6.19 (2.38–16.13)		5.55 (2.11–14.61)		5.40 (2.04–14.27)	
F	6.63 (2.43–18.05)		6.46 (2.34–17.83)		5.90 (2.12–16.42)	
G	7.30 (2.71–19.63)		6.51 (2.40–17.67)		6.29 (2.30–17.18)	
H	7.74 (2.94–20.41)		7.28 (2.74–19.33)		6.45 (2.41–17.29)	
I	9.22 (3.45–24.69)		8.91 (3.31–24.04)		8.67 (3.20–23.52)	
J	11.35 (3.37–38.15)		7.06 (2.01–24.76)		7.28 (2.07–26.65)	
K	14.05 (5.22–37.77)		8.20 (2.96–22.70)		7.73 (2.76–21.63)	
Refused any questions during in-person enrollment questionnaire^c^		0.004				
No	Reference					
Yes	7.93 (1.97–31.97)					
Answered ”Don’t know” to any questions during enrollment in-person questionnaire^c^		0.01				
No	Reference					
Yes	1.82 (1.14–2.92)					
Refused/missed any questions during enrollment self-administered questionnaires^c^		<0.0001		<0.0001		<0.0001
No	Reference		Reference		Reference	
Yes	2.48 (1.81–3.41)		1.97 (1.40–2.76)		2.09 (1.48–2.95)	
Scheduled next interview phone call during intake appointment^c^		0.001				
No	1.77 (1.25–2.50)					
Yes	Reference					
Coordinator worked in a prior polyp prevention trial^c^		0.01				
No	Reference					
Yes	0.61 (0.41–0.90)					
Preference: If you could choose, which kind of pill would you like to receive during the study?		0.09				
Calcium + vitamin D	Reference					
Calcium only	1.86 (1.01–3.43)					
Vitamin D only	1.84 (0.97–3.46)					
Placebo	0.56 (0.13–2.36)					
Don’t know/refused	1.25 (0.88–1.77)					
Had a perceived toxicity during run-in						<0.0001
No					Reference	
Yes					12.21 (5.08–29.33)	

^a^Included were all variables that had *p* < 0.1 from Table 1 and baseline factors from Table 2

^b^Final model plus perceived toxicity, the post-enrollment factor from Table 2

^c^Not a priori potential predictors

**Table 4:** Logistic regression models of run-in failure: full factorial study females

	Unadjusted OR (95 % CI)^a^	*p*	Final model: adjusted OR (95 % CI) C index = 0.62	*p*	Final model + perceived toxicity adjusted OR (95 % CI)^b^ C index = 0.66	*p*
Total participants			*N* = 301		*N* =301	
Race		0.06				
White	Reference					
Black	0.92 (0.34–2.52)					
Other	2.85 (0.50–16.13)					
Unknown/refused	4.75 (1.38–16.38)					
Hispanic ethnicity		0.05				
No	Reference					
Yes	3.07 (0.98–9.59)					
Marital status		0.04				
Married/cohabitating	Reference					
Single	1.95 (1.04–3.66)					
Education		0.08				
Did not graduate high school	2.75 (1.05–7.23)					
Graduated high school	1.69 (0.79–3.63)					
Any college education	Reference					
Smoking status		0.07				
Never	Reference					
Former	1.80 (0.91–3.56)					
Current	2.55 (1.06–6.17)					
Number of prescription medications		0.03		0.03		0.03
0	Reference		Reference		Reference	
1	0.43 (0.14–1.30)		0.43 (0.14–1.30)		0.40 (0.13–1.26)	
2	0.21 (0.05–0.87)		0.21 (0.05–0.87)		0.21 (0.05–0.87)	
3+	0.99 (0.41–2.39)		0.99 (0.41–2.39)		0.97 (0.39–2.38)	
Experienced muscular pain in last year		0.07				
None	Reference					
Some	0.39 (0.11–1.31)					
Severe	3.66 (0.79–16.96)					
Experienced muscular weakness in last year		0.10				
None/some	Reference					
Severe	5.32 (0.73–38.71)					
Refused any questions during enrollment self-administered questionnaires^c^		0.03				
No	Reference					
Yes	2.41 (1.11–5.27)					
Had a perceived toxicity during run-in						0.001
No					Reference	
Yes					12.26 (2.79–54.00)	

^a^Included were all variables that had *p* < 0.1 from Table 1 and baseline factors from Table 2

^b^Final model plus perceived toxicity, the post-enrollment factor from Table 2

^c^Not a priori potential predictors

**Table 5:** Logistic regression models of run-in failure: two-group randomization females

	Unadjusted OR (95 % CI)^a^	*p*	Final model: adjusted OR (95 % CI) C index = 0.79	*p*	Final model + perceived toxicity adjusted OR (95 % CI)^b^ C index = 0.81	*p*
Total participants			*N* = 659		*N* = 659	
Race		0.01				
White	Reference					
Black	2.44 (1.29–4.62)					
Other	0.48 (0.06–3.66)					
Unknown/refused	2.50 (1.21–5.17)					
Marital status		0.04				
Married/cohabitating	Reference					
Single	1.66 (1.03–2.69)					
Education		0.03				
Did not graduate high school	2.38 (1.21–4.69)					
Graduated high school	1.52 (0.82–2.83)					
Any college education	Reference					
Taking a multivitamin^c^		0.001		0.002		0.004
No	Reference		Reference		Reference	
Yes	0.44 (0.27–0.71)		0.44 (0.26–0.75)		0.45 (0.26–0.77)	
Taking calcium supplements^c^		0.001				
No	Reference					
Yes	0.45 (0.28–0.73)					
Taking vitamin D supplements^c^						
No	Reference	0.0003				
Yes	0.31 (0.16–0.58)					
SF-36 mental component summary measure (per 5 units)	0.81 (0.70–0.94)	0.01				
SF-36 physical component summary measure (per 5 units)	0.75 (0.65–0.86)	<0.0001	0.73 (0.62–0.86)	0.0002	0.70 (0.58–0.83)	<0.0001
Study center		<0.0001		<0.0001		<0.0001
A	Reference		Reference		Reference	
B	1.51 (0.29–7.74)		0.86 (0.14–5.42)		1.00 (0.15–6.56)	
C	1.27 (0.21–7.90)		1.39 (0.22–8.82)		1.68 (0.25–11.13)	
D	1.33 (0.22–8.28)		1.07 (0.17–6.85)		1.23 (0.19–8.17)	
E	2.24 (0.52–9.72)		1.84 (0.42–8.18)		1.88 (0.40–8.85)	
F	14.67 (4.16–51.74)		13.17 (3.65–47.58)		14.37 (3.76–55.00)	
G	1.85 (0.40–8.56)		2.02 (0.43–9.52)		1.97 (0.38–10.30)	
H	6.31 (1.68–23.69)		4.90 (1.28–18.80)		5.32 (1.31–21.53)	
I	5.50 (1.52–19.88)		5.58 (1.51–20.68)		6.18 (1.58–24.18)	
J	6.37 (0.94–43.30)		5.36 (0.73–39.45)		6.63 (0.85–51.46)	
K	7.72 (2.12–28.14)		4.12 (1.06–15.97)		3.12 (0.75–13.02)	
Colonoscopy surveillance interval		0.004		0.03		0.02
3 years	Reference		Reference		Reference	
5 years	2.08 (1.27–3.41)		1.91 (1.08–3.37)		2.06 (1.13–3.74)	
Refused any questions during enrollment intake questionnaire^d^		0.01				
No	Reference					
Yes	22.14 (2.28–215.43)					
Answered ”Don’t know” to any questions during enrollment intake questionnaire^d^		0.05				
No	Reference					
Yes	1.85 (1.00–3.43)					
Refused any questions during enrollment self-administered questionnaires^d^		0.002				
No	Reference					
Yes	2.49 (1.42–4.38)					
Counseled at baseline to change diet^d^		0.07				
No	Reference					
Yes	3.71 (0.91–15.12)					
Improve mood		0.06				
Very/somewhat likely	Reference					
Don’t know	0.54 (0.32–0.92)					
Very/somewhat unlikely	0.65 (0.33–1.29)					
Cause constipation		0.10				
Very/somewhat likely	Reference					
Don’t know	0.55 (0.31–0.96)					
Very/somewhat unlikely	0.61 (0.33–1.12)					
Had a perceived toxicity during the run-in period						<0.0001
No					Reference	
Yes					29.02 (6.83–123.33)	

^a^Included were all variables that had *p* < 0.1 from Table 1 and baseline factors from Table 2

^b^Final model plus perceived toxicity, the post-enrollment factor from Table 2

^c^Baseline multivitamin, calcium, and vitamin D supplement use were collinear variables; the first was included in the model

^d^Not a priori potential predictors

Perceived toxicities (PTs) were reported during run-in by 34 (1.8 %) participants in the full factorial randomization and by 12 (1.8 %) women in the two-group randomization. Of these 46 participants, 29 (63 %) became run-in failures, representing 12-fold increased odds of RIF in the full factorial randomization groups and 29-fold in the two-group randomization. Inclusion of PTs in the final models did not substantially alter the estimates for the other RIF predictors (Tables 3, 4, 5).

Study center was included in the final multivariable model for men (Table 3) and for women in the two-arm randomization (Table 5). When center was omitted from the model of RIF in men, two factors became significant: the odds of failing run-in were higher among men who had not scheduled a time for the next phone call before they left the enrollment interview (OR 1.61; 95 % CI 1.11–2.33), and lower among men enrolled by a coordinator who had worked in a prior polyp prevention study (OR 0.64; 95 % CI 0.42–0.97) (see Additional file 3). Omission of center from the model also led to a larger odds ratio for the lowest education category (3.25; 95 % CI 1.92–5.49 from 2.77; 95 % CI 1.58–4.85). Variable selection for the model for women in the two-arm randomization was not affected by exclusion of center (see Additional file 4).

In analyses by center, the risk of run-in was inversely correlated with three measures of post-randomization adherence based on pill-taking and/or endpoint ascertainment, but none of these associations reached statistical significance (see Additional files 5, 6, and **7**).

## Discussion

In this study, we analyzed data from the first 3 months of a long-term chemoprevention trial to identify baseline predictors of voluntary run-in failure (RIF) in three groups of participants. In all, 12 % of participants failed run-in, and this loss before randomization represents considerable effort that was invested specifically to improve long-term trial efficiency. Our analyses uncovered differences in the factors associated with RIF in the three groups, even though they experienced fundamentally the same trial conditions. RIF risk in men was primarily associated with sociodemographic characteristics, but the key drivers in women were health-related factors. There were further differences in RIF predictors among women in the full factorial and two-group randomization protocols. The former group experienced a true, single-blind, placebo run-in, whereas the latter received calcium during run-in; thus, their single-blind run-in was potentially influenced by the physiological effects of calcium as well as any health beliefs related to their preference for calcium supplementation. Our results illustrate the difficulties in defining a simple, generalizable set of risk factors to identify participants at risk of RIF. While one might expect to see different factors affecting RIF in trials that involve different diseases, interventions, and outcomes, here we see different multivariable models in men and women within the same trial.

In the two larger groups, study center was associated with an 8- to 13-fold variation in odds of RIF, even after adjustment for other factors. This substantial effect may reflect differences in the methods used by study staff at each center, or it may be due to residual confounding by medical, educational, cultural, or other characteristics of the participants. With the available data, we could not identify differences in methods between centers. However, in post hoc analyses designed to explore the substantial heterogeneity in RIF by center, we found that building the model without center led, among men, to stronger associations between run-in failure and coordinator experience, timely scheduling of the next interview, and participant education. Center may represent a mix of factors including participant and coordinator characteristics, participants’ uncertainty about their commitment, and competing constraints on their time (e.g., by employment). Center has been associated with RIF risk in other settings [6, 7], and this association may be worth exploring in future trials. Will a center with more RIFs subsequently have better adherence and endpoint ascertainment because participants were more stringently selected, or does a high RIF rate indicate a local problem that will persist throughout the study? Our exploratory post hoc data hinted that centers with more RIFs had lower rates of post-randomization adherence and trial completion; this might suggest local differences by center, e.g., in study methods, or in intrinsic differences among the participants who enrolled in each region.

Among men, those who were younger, single, and had not graduated high school had the greatest risk of failing run-in. One possible explanation is that those who were working had less time to commit to the study than retired participants, but we did not collect employment data. In addition, men had twice the risk of RIF if they overlooked or refused to answer one or more questions in two self-administered questionnaires (SAQs). This finding might be explained by an association between perfect completion of the SAQ and an individual’s motivations underlying trial participation. However, an alternative explanation is differences in quality checking by study staff; those who more effectively identified missing SAQ questions and had participants correct them may have also been better at motivating enrollees during enrollment and run-in. With our data, we could not distinguish these two possibilities. The first suggests that a SAQ could be developed to help identify individuals at risk of run-in failure and target them for intervention. The second could be addressed via improvements in staff training and motivational protocols.

In women, health-related factors tended to be associated with RIF. In the full factorial randomization, RIF was most frequent among women taking no prescription medications at baseline. Among women in the two-group randomization, RIF was less common among women who took vitamin supplements at baseline, had better SF-36 physical health scores, and a 3-year (rather than a 5-year) trial participation, which represents not only a shorter commitment to trial participation, but also higher risk adenomas that require more intensive follow-up. We found no convincing evidence that the successful negotiation of run-in was associated with participants’ expectations of health benefits from the agents, which tablets they would prefer if given the choice, or which agents they guessed they would be given for the study. Other studies have found that trial participation is associated with altruistic factors [8–11], so it is possible that individuals with stronger feelings about the study agents were less likely to enroll in the first place.

Predictors of RIF have varied in previous trials. RIF was associated with lower Karnofsky performance score and lower education level in a head and neck cancer chemoprevention trial [12], and with younger age, not working, and smoking, in a trial of pregnant women to prevent adverse neonatal outcomes [7]. Interestingly, in the latter study, the proportions of RIFs ranged from 20–40 % in five clinics but was lowest in one clinic where participants were told they would receive sugar pills during run-in. Another trial of motivational interventions to reduce blood pressure found no significant characteristics of RIFs, but the sample was small [1].

In our trial and others, the purpose of the adherence run-in is to help researchers identify and randomize the participants most likely to follow trial protocol. Its potential advantages include the retention of good long-term adherers [13], improved efficiency [14], improved internal validity in the estimation of efficacy, and greater statistical precision [15]. There is certainly evidence that removing poor adherers can change a study’s effect estimates. For example, in a trial of lovastatin to reduce cholesterol levels, Davis et al. found that participants who would have failed run-in had an adherence criterion been applied, experienced 17.8 % smaller cholesterol reductions than more adherent participants [6]. By retaining these individuals in the trial, the proportion of less educated participants was increased (improving generalizability), but the overall measure of lovastatin’s effectiveness was 5.2 % lower than it would otherwise have been. Pablos-Mendez discussed two comparable trials of aspirin to prevent myocardial infarction [2]. The first trial, in American physicians, included a run-in and reported 90 % adherence over 5 years and a significant risk reduction of 44 % [16]. The other trial, in British physicians, did not include a run-in, and reported adherence of 70 % over 6 years and a non-significant risk reduction of 3 % [17]. These findings support the use of run-in to improve adherence, provided that the goal is to estimate efficacy rather than effectiveness.

One concern about the adherence run-in is that individuals who adhere poorly to treatment are very different than those who adhere well, with respect to health and other characteristics; poor adherers have worse health outcomes during clinical trials independent of assignment to the active or placebo group [18–23], and their exclusion may therefore reduce generalizability. Further, improvements in efficiency offered by the run-in may be attenuated if the exclusion criteria misclassify individuals who would have gone on to complete the trial successfully [14]. Although early trial adherence and longer term adherence tend to be highly correlated [7], one study showed that this was true for less educated participants but not for more educated ones [6]. The implication is that exclusion of better educated participants who adhere poorly during run-in may be relatively inefficient because their longer term adherence is less accurately predicted. However, although the factors associated with adherence in different settings have been studied extensively [24, 25], RIF is not simply an adherence issue; it also gives participants an opportunity to reconsider their enrollment (“buyer’s remorse”). In one study, when patients were interviewed within a month of enrollment in a variety of clinical trials, 16 (12 %) had already considered dropping out, and 4 of those continued because of a sense of obligation, despite a preference to withdraw [26]. In our study, 21 % of RIFs (4 % of enrollees medically eligible for randomization) clearly stated that they did not want to continue participating, and a further 12 % were uncooperative or could not be contacted. But among the two-thirds of RIFs we attributed to poor adherence, ambivalence towards trial participation may have been the underlying cause of that poor adherence in some cases. It is also possible that the $100 incentive payment was the primary motivation for enrollment; this may account for some early dropouts.

Future studies of run-in might collect more granular data to distinguish poor adherence from disinclination to continue in the trial. Potential strategies to address both problems may include increased communication with participants during run-in to elicit questions and concerns, motivate participants in their pill-taking routine, and develop a rapport that encourages individuals to stay in the trial. One option might be a two-stage run-in. For example, in our trial, participants could have been telephoned one week after enrollment to assess early adherence, identify cases of “buyer’s remorse,” and provide explanations and motivational counseling where appropriate. After 3 months, persistent non-adherers could be excluded from randomization as usual, and randomization could be stratified according to whether motivational counseling was initiated, to assess the impact of the strategy by subgroup. This approach could be extended to study the ideal frequency of participant contact or counseling needed to retain promising participants but not reluctant enrollees with poor long-term prospects of adherence.

Forty-six participants (2 % of enrollees and 15 % of RIFs) contacted their coordinator to report perceived toxicities (PTs) during run-in, and almost two-thirds of those became RIFs. In the full factorial randomization, participants received placebo during run-in and PTs were associated with a 12-fold increased odds of RIF. In the two-group randomization, women receiving calcium and reporting a PT experienced a 29-fold increase in odds of RIF. However, the risk of PT was similar in those given placebo or calcium during run-in. When a participant develops any new symptom by chance during run-in, they may attribute it to the study intervention; alternatively, the PT may be a nocebo phenomenon arising from expectations of an adverse effect upon starting a new treatment. Up to a quarter of patients in the placebo group in previous trials have reported symptoms after randomization, with a wide range of frequency across trials [27–29]. Some trials deliberately exclude participants who show a placebo response (or a coincident improvement in health) during run-in, but they may be criticized for poorer generalizability [4]. What we observed was an increased tendency for participants with a nocebo response (or coincidental development of symptoms) to fail run-in. Both types of exclusion involve a change in health while taking placebo: the first, determined by the investigator, excludes those with beneficial changes in health while taking placebo, while the second type, determined by the participant, excludes those with adverse changes in health. Both are likely to affect generalizability. Although PTs were very strongly associated with RIF, it is unclear how to address this, because if the blind must be maintained, participants cannot be told that they were taking placebo during run-in.

This study was limited by differences in sample size in the three subgroups, which will have affected the power to consistently detect factors with common specific effect sizes and meant that our multivariable modeling did not directly compare RIF rates in men and women. In addition, we chose to only consider main effects in our analyses and did not model any interactions between potential predictor variables. A further limitation was our use of self-reported adherence via tablet counts, which, although superior to some methods, is still subject to misclassification [30]. Our study was a relatively long-term (3- to 5-year) trial using agents (calcium and vitamin D) that are generally thought to have few, if any, adverse side effects. As a chemoprevention trial, it recruited individuals with a specific interest in preventive health strategies, and our study was also limited to adults aged 45–75 in good general health. The study may therefore have limited generalizability to other types of trials, although this would be more of a concern if we had found a consistent set of RIF predictors in the three subgroups and were proposing to extend our findings to diverse trial settings.

## Conclusions

The lack of a clear, single set of predictors of run-in failures among our three subgroups suggests that the search for general predictors of run-in failure in trials will not be straightforward. However, substantially different risks associated with study center and missing self-administered questionnaire data reflect opportunities for further study with the goal of identifying interventions that might improve trial efficiency and retention. Perceived toxicities during a placebo run-in represent the strongest risk factor for run-in failure, but perhaps are the most difficult to address in a blinded study. The loss of 12 % of participants due to voluntary factors represents considerable expense as well as loss of generalizability, and the search for a balance between optimal efficiency and generalizability via the adherence run-in deserves further attention.

## Abbreviations

CI, confidence interval; MCS, mental component summary measure (SF-36); OR, odds ratio; PCS, physical component summary measure (SF-36); PT, perceived toxicity; RIF, run-in failure; SAQ, self-administered questionnaire

## Additional files

Additional file 1: Institutional Review Boards that approved the trial at each study center. (DOCX 14 kb) Additional file 2: CONSORT 2010 checklist of information to include when reporting a randomised trial. (DOC 213 kb) Additional file 3: Logistic regression models of voluntary run-in failure, excluding study center: full factorial study males (DOCX 20 kb) Additional file 4: Logistic regression models of run-in failure, excluding study center: two-group randomization females. (DOCX 19 kb) Additional file 5: Run-in failure and measures of post-randomization adherence, by center. Successful voluntary completion (%) is defined for each center as the number of participants with an end-of-treatment colonoscopy result and mean self-reported adherence ≥50 % throughout the trial (3–5 years), divided by the number of participants medically eligible to complete the trial. The measure excludes participants who involuntarily dropped out of the study or stopped taking pills for valid medical reasons. The correlation with RIF (%) measured by Kendall’s tau-b is –0.22 (95 % CI –0.81 to 0.37; *p* = 0.40). (PDF 43 kb) Additional file 6: Run-in failure and measures of post-randomization adherence, by center. Outcome data provision rate (%) is defined for each center as the number of participants with an end-of-treatment colonoscopy result, divided by the number of participants medically eligible to complete the trial. The measure excludes participants who involuntarily dropped out of the study or stopped taking pills for valid medical reasons. The correlation with RIF (%) measured by Kendall’s tau-b is –0.27 (95 % CI –0.68 to 0.13; *p* = 0.28). (PDF 43 kb) Additional file 7: Run-in failure and measures of post-randomization adherence, by center. Average adherence (%) is defined for each center throughout the trial (3–5 years) as the self-reported number of pills taken by all participants medically eligible to take study pills throughout the trial, divided by the maximum number of pills that they should have taken. The measure excludes participants who involuntarily dropped out of the study or stopped taking pills for valid medical reasons. The correlation with RIF (%) measured by Kendall’s tau-b is –0.31 (95 % CI –0.78 to 0.16; *p* = 0.22). (PDF 45 kb)
