# Supplementary material for: Randomized controlled trials: who fails run-in?
Source: Trials. 2016 Jul 29;17:374. doi: 10.1186/s13063-016-1451-9 (PMC4966775; doi:10.1186/s13063-016-1451-9)
Supplement: Additional file 1: — Institutional Review Boards that approved the trial at each study center. (DOCX 14 kb) [file 13063_2016_1451_MOESM1_ESM.docx]

Institutional Review Boards that approved the trial at each study center

Dartmouth College Committee for the Protection of Human Subjects, University of Southern California IRB, Kaiser Permanente IRB, Colorado Multiple Institutional Review Board, Emory University IRB, Atlanta Veterans Affairs Medical Center (VAMC) IRB, University of Iowa IRB, University of Minnesota IRB, Minneapolis VAMC IRB, Portsmouth Regional Hospital IRB, Concord Hospital IRB, Maine Medical Center IRB, White River Junction VAMC IRB, University of North Carolina IRB, Cleveland Clinic IRB, University of Puerto Rico IRB, San Juan VAMC IRB, Palmetto Health IRB, University of Texas/MD Anderson Cancer Center IRB.

.
