# Supplementary material for: Randomized controlled trials: who fails run-in?
Source: Trials. 2016 Jul 29;17:374. doi: 10.1186/s13063-016-1451-9 (PMC4966775; doi:10.1186/s13063-016-1451-9)
Supplement: Additional file 3: — Logistic regression models of voluntary run-in failure, excluding study center: full factorial study males (DOCX 20 kb) [file 13063_2016_1451_MOESM3_ESM.docx]

|  | Unadjusted OR (95% CI)^a^ | p | Final model: adjusted  OR (95% CI)  C Index =0.69 | p | Final model + perceived toxicity  adjusted  OR (95% CI)^b^  C Index =0.72 | p |
| --- | --- | --- | --- | --- | --- | --- |
| Total participants |  |  | N=1604 |  | N=1604 |  |
| Age (per 5 years) | 0.87 (0.78-0.97) | 0.01 | 0.86 (0.76-0.96) | 0.01 | 0.85 (0.75-0.96) | 0.01 |
| Race |  | 0.01 |  |  |  |  |
| White | reference |  |  |  |  |  |
| Black | 1.82 (1.10-3.01) |  |  |  |  |  |
| Other | 1.29 (0.60-2.76) |  |  |  |  |  |
| Unknown/refused | 2.21 (1.20-4.07) |  |  |  |  |  |
| Hispanic ethnicity |  | <0.0001 |  |  |  |  |
| No | reference |  |  |  |  |  |
| Yes | 2.62 (1.62-4.22) |  |  |  |  |  |
| Marital status |  | 0.001 |  | 0.005 |  | 0.002 |
| Single | 1.89 (1.29-2.76) |  | 1.77 (1.19-2.63) |  | 1.87 (1.25-2.79) |  |
| Married/cohabitating | reference |  | reference |  | reference |  |
| Education |  | <0.0001 |  | <0.0001 |  | 0.0001 |
| Did not graduate high school | 3.56 (2.18-5.81) |  | 3.25 (1.92-5.49) |  | 3.03 (1.77-5.18) |  |
| Graduated high school | 0.92 (0.57-1.50) |  | 0.88 (0.53-1.46) |  | 0.85 (0.51-1.41) |  |
| Any college education | reference |  | reference |  | reference |  |
| Taking a multivitamin |  | 0.04 |  |  |  |  |
| No | reference |  |  |  |  |  |
| Yes | 0.72 (0.53-0.99) |  |  |  |  |  |
| Taking calcium supplements |  | 0.07 |  |  |  |  |
| No | reference |  |  |  |  |  |
| Yes | 0.46 (0.20-1.05) |  |  |  |  |  |
| Taking vitamin D supplements |  | 0.06 |  |  |  |  |
| No | reference |  |  |  |  |  |
| Yes | 0.25 (0.06-1.03) |  |  |  |  |  |
| Experienced muscular weakness in last year |  | 0.09 |  |  |  |  |
| None | reference |  |  |  |  |  |
| Some | 1.81 (0.95-3.45) |  |  |  |  |  |
| Severe | 2.22 (0.61-8.03) |  |  |  |  |  |
| SF36 mental score (per 5 units) | 0.90 (0.80-1.01) | 0.08 |  |  |  |  |
| Refused any questions during in-person enrollment questionnaire^c^ |  | 0.004 |  |  |  |  |
| No | reference |  |  |  |  |  |
| Yes | 7.93 (1.97-31.97) |  |  |  |  |  |
| Answered ‘Don’t know’ to any questions during enrollment in-person questionnaire^c^ |  | 0.01 |  |  |  |  |
| No | reference |  |  |  |  |  |
| Yes | 1.82 (1.14-2.92) |  |  |  |  |  |
| Refused/missed any questions during enrollment self administered questionnaires^c^ |  | <0.0001 |  | <0.0001 |  | <0.0001 |
| No | reference |  | reference |  | reference |  |
| Yes | 2.48 (1.81-3.41) |  | 2.31 (1.66-3.20) |  | 2.44 (1.75-3.41) |  |
| Scheduled next interview phone call during intake appointment^c^ |  | 0.001 |  | 0.01 |  | 0.02 |
| No | 1.77 (1.25-2.50) |  | 1.61 (1.11-2.33) |  | 1.56 (1.07-2.28) |  |
| Yes | reference |  | reference |  | reference |  |
| Coordinator worked in a prior PPSG study^c^ |  | 0.01 |  | 0.03 |  | 0.03 |
| No | reference |  | reference |  | reference |  |
| Yes | 0.61 (0.41-0.90) |  | 0.64 (0.42-0.97) |  | 0.63 (0.41-0.96) |  |
| Preference: If you could choose, which kind of pill would you like to receive during the study? |  | 0.09 |  |  |  |  |
| Calcium + vitamin D | reference |  |  |  |  |  |
| Calcium only | 1.86 (1.01-3.43) |  |  |  |  |  |
| Vitamin D only | 1.84 (0.97-3.46) |  |  |  |  |  |
| Placebo | 0.56 (0.13-2.36) |  |  |  |  |  |
| Don’t know/refused | 1.25 (0.88-1.77) |  |  |  |  |  |
| Had a perceived toxicity during run-in |  |  |  |  |  | <0.0001 |
| No |  |  |  |  | reference |  |
| Yes |  |  |  |  | 12.26 (5.25-28.61) |  |

^a^ Included were all variables that had p<0.1 from Table 1 or baseline factors from Table 2

^b^ Final model plus Perceived Toxicity, the post enrollment factor from Table 2

^c^ Not *a priori* potential predictors

The model in full factorial study females was unaffected by exclusion of center, and is not presented
