# Supplementary material for: Randomized controlled trials: who fails run-in?
Source: Trials. 2016 Jul 29;17:374. doi: 10.1186/s13063-016-1451-9 (PMC4966775; doi:10.1186/s13063-016-1451-9)
Supplement: Additional file 4: — Logistic regression models of run-in failure, excluding study center: two-group randomization females. (DOCX 19 kb) [file 13063_2016_1451_MOESM4_ESM.docx]

|  | Unadjusted OR (95% CI)^a^ | p | Final model: adjusted  OR (95% CI)  C Index = 0.69 | p | Final model + perceived toxicity  adjusted  OR (95% CI)^b^  C Index =0.72 | p |
| --- | --- | --- | --- | --- | --- | --- |
| Total participants |  |  | N=659 |  | N=659 |  |
| Race |  | 0.01 |  |  |  |  |
| White | Reference |  |  |  |  |  |
| Black | 2.44 (1.29-4.62) |  |  |  |  |  |
| Other | 0.48 (0.06-3.66) |  |  |  |  |  |
| Unknown/refused | 2.50 (1.21-5.17) |  |  |  |  |  |
| Marital Status |  | 0.04 |  |  |  |  |
| Married/cohabitating | Reference |  |  |  |  |  |
| Single | 1.66 (1.03-2.69) |  |  |  |  |  |
| Education |  | 0.03 |  |  |  |  |
| Did not graduate high school | 2.38 (1.21-4.69) |  |  |  |  |  |
| Graduated high school | 1.52 (0.82-2.83) |  |  |  |  |  |
| Any college education | Reference |  |  |  |  |  |
| Taking a multivitamin^c^ |  | 0.001 |  | 0.001 |  | 0.001 |
| No | Reference |  | Reference |  | Reference |  |
| Yes | 0.44 (0.27-0.71) |  | 0.44 (0.27-0.71) |  | 0.44 (0.27-0.73) |  |
| Taking calcium supplements^c^ |  | 0.001 |  |  |  |  |
| No | Reference |  |  |  |  |  |
| Yes | 0.45 (0.28-0.73) |  |  |  |  |  |
| Taking vitamin D supplements^c^ |  |  |  |  |  |  |
| No | Reference | 0.0003 |  |  |  |  |
| Yes | 0.31 (0.16-0.58) |  |  |  |  |  |
| SF36 mental score (per 5 units) | 0.81 (0.70-0.94) | 0.01 |  |  |  |  |
| SF36 physical score (per 5 units) | 0.75 (0.65-0.86) | <0.0001 | 0.74 (0.64-0.86) | 0.0001 | 0.70 (0.58-0.83) | <0.0001 |
| Colonoscopy surveillance interval |  | 0.004 |  | 0.004 |  | 0.004 |
| 3 year | Reference |  | Reference |  | Reference |  |
| 5 year | 2.08 (1.27-3.41) |  | 2.14 (1.28-3.57) |  | 2.22 (1.30-3.79) |  |
| Refused any questions during enrollment – intake questionnaire^d^ |  | 0.01 |  |  |  |  |
| No | Reference |  |  |  |  |  |
| Yes | 22.14 (2.28-215.43) |  |  |  |  |  |

| Answered ‘Don’t know’ to any questions during enrollment – intake questionnaire^d^ |  | 0.05 |  |  |  |  |
| --- | --- | --- | --- | --- | --- | --- |
| No | Reference |  |  |  |  |  |
| Yes | 1.85 (1.00-3.43) |  |  |  |  |  |
| Refused any questions during enrollment – self-administered questionnaires^d^ |  | 0.002 |  |  |  |  |
| No | Reference |  |  |  |  |  |
| Yes | 2.49 (1.42-4.38) |  |  |  |  |  |
| Counseled at baseline to change diet |  | 0.07 |  |  |  |  |
| No | Reference |  |  |  |  |  |
| Yes | 3.71 (0.91-15.12) |  |  |  |  |  |
| Improve mood |  | 0.06 |  |  |  |  |
| Very/somewhat likely | Reference |  |  |  |  |  |
| Don’t know | 0.54 (0.32-0.92) |  |  |  |  |  |
| Very/somewhat unlikely | 0.65 (0.33-1.29) |  |  |  |  |  |
| Cause constipation |  | 0.10 |  |  |  |  |
| Very/somewhat likely | Reference |  |  |  |  |  |
| Don’t know | 0.55 (0.31-0.96) |  |  |  |  |  |
| Very/somewhat unlikely | 0.61 (0.33-1.12) |  |  |  |  |  |
| Had a Perceived Toxicity during the run-in period |  |  |  |  |  | <0.0001 |
| No |  |  |  |  | Reference |  |
| Yes |  |  |  |  | 28.70 (7.23, 113.90) |  |

^a^ Included were all variables that had p<0.1 from Table 1 or baseline factors from Table 2

^b^ Final model plus Perceived Toxicity, the post enrollment factor from Table 2

^c^ Baseline multivitamin, calcium and vitamin D supplement use were collinear variables; the first was included in the model

^d^ Not *a priori* potential predictors
