# Supplementary figures and images for: Randomized controlled trials: who fails run-in?
Source: Trials. 2016 Jul 29;17:374. doi: 10.1186/s13063-016-1451-9 (PMC4966775; doi:10.1186/s13063-016-1451-9)

a. Successful completion rate, by center

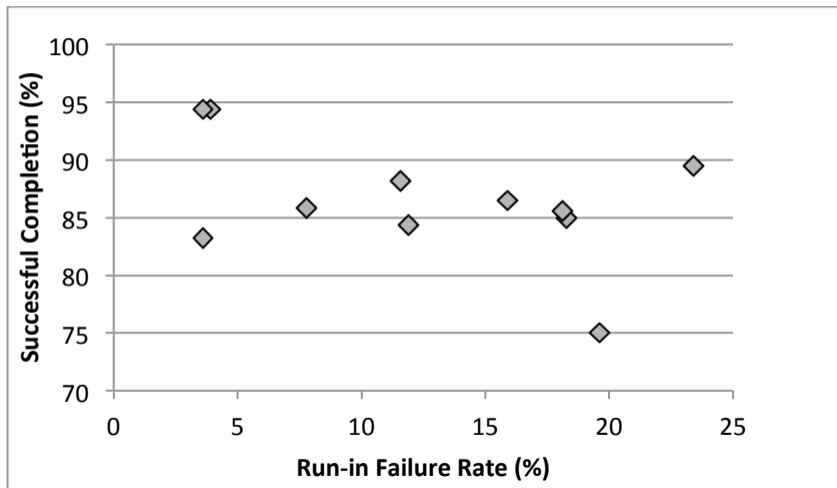

Supplement: Additional file 5: — Run-in failure and measures of post-randomization adherence, by center. Successful voluntary completion (%) is defined for each center as the number of participants with an end-of-treatment colonoscopy result and mean self-reported adherence ≥50 % throughout the trial (3–5 years), divided by the number of participants medically eligible to complete the trial. The measure excludes participants who involuntarily dropped out of the study or stopped taking pills for valid medical reasons. The correlation with RIF (%) measured by Kendall’s tau-b is –0.22 (95 % CI –0.81 to 0.37; p = 0.40). (PDF 43 kb) [file 13063_2016_1451_MOESM5_ESM.pdf]

b. Trial endpoint data obtained at three to five years, by center

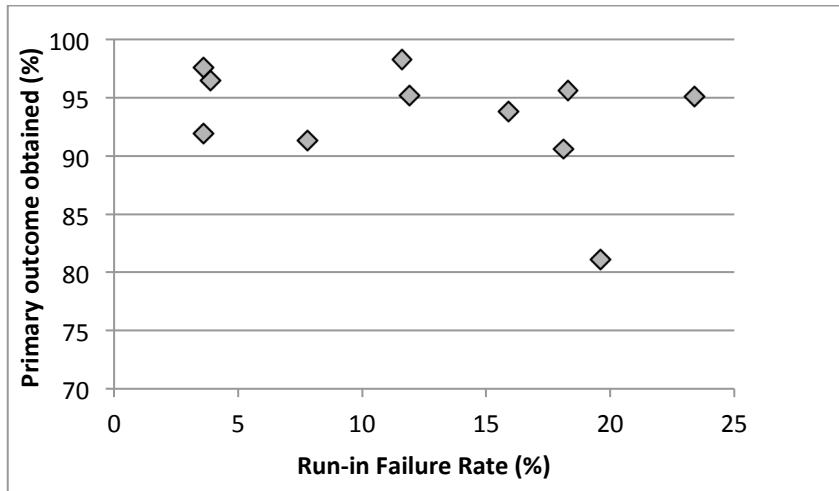

Supplement: Additional file 6: — Run-in failure and measures of post-randomization adherence, by center. Outcome data provision rate (%) is defined for each center as the number of participants with an end-of-treatment colonoscopy result, divided by the number of participants medically eligible to complete the trial. The measure excludes participants who involuntarily dropped out of the study or stopped taking pills for valid medical reasons. The correlation with RIF (%) measured by Kendall’s tau-b is –0.27 (95 % CI –0.68 to 0.13; p = 0.28). (PDF 43 kb) [file 13063_2016_1451_MOESM6_ESM.pdf]

c. Average adherence between randomization and end of trial, by center

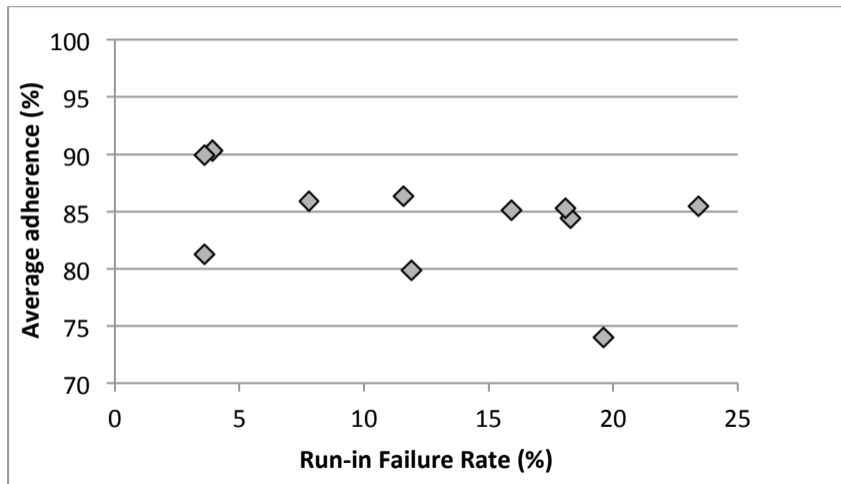

Supplement: Additional file 7: — Run-in failure and measures of post-randomization adherence, by center. Average adherence (%) is defined for each center throughout the trial (3–5 years) as the self-reported number of pills taken by all participants medically eligible to take study pills throughout the trial, divided by the maximum number of pills that they should have taken. The measure excludes participants who involuntarily dropped out of the study or stopped taking pills for valid medical reasons. The correlation with RIF (%) measured by Kendall’s tau-b is –0.31 (95 % CI –0.78 to 0.16; p = 0.22). (PDF 45 kb) [file 13063_2016_1451_MOESM7_ESM.pdf]
